# COVID-19 Pandemic-Related Depression and Insomnia among Psychiatric Patients and the General Population

**DOI:** 10.3390/jcm10153425

**Published:** 2021-07-31

**Authors:** Anna Klimkiewicz, Joanna Jasionowska, Adrianna Schmalenberg, Jakub Klimkiewicz, Agata Jasińska, Andrzej Silczuk

**Affiliations:** 1 Department of Psychiatry, Medical University of Warsaw, Nowowiejska St. 27, 00-665 Warsaw, Poland; anna.klimkiewicz@wum.edu.pl; 2 Psychomedic Clinic, Jastrzebowskiego St. 24, 02-783 Warsaw, Poland; adriannaschm@gmail.com; 3 Nowowiejski Psychiatric Hospital, Nowowiejska St. 27, 00-665 Warsaw, Poland; joanna.j@neostrada.pl; 4 Department of Psychology, SWPS University, Chodakowska St. 19/31, 03-815 Warsaw, Poland; 5 Military Institute of Medicine, Szaserow St. 128, 04-141 Warsaw, Poland; 6 Department of Science and Technology Transfer, Medical University of Warsaw, Żwirki i Wigury St. 61, 02-091 Warsaw, Poland; agata.jasinska@wum.edu.pl; 7 Institute of Psychiatry and Neurology, Addiction Prevention and Treatment Team, Sobieskiego St. 9, 02-957 Warsaw, Poland; asilczuk@ipin.edu.pl

**Keywords:** COVID-19, insomnia, depression, social support, economic crisis, employment

## Abstract

Background: The COVID-19 pandemic and limited access to healthcare professionals pose a serious risk of worsening mental conditions. This study was designed to examine the changes in symptoms of insomnia and depression during the pandemic as compared to before the pandemic, as well as the factors correlated with abovementioned mental state deterioration. Methods: The study was conducted from 1 April to 15 May 2020, on 212 psychiatric outpatients and 207 healthy controls. Participants completed a survey focused on symptoms during and prior to COVID-19 (the Beck Depression Inventory, the Athens Insomnia Scale). The following correlations were analyzed: demographics, social support, work status, income, and possible participants’ and their relatives’ COVID-19 diagnoses. Results: Insomnia and depression severity intensified during the pandemic in both groups and were associated with age, gender, education, employment, and financial status. No correlations between social support nor becoming sick with COVID-19 and insomnia or depression were observed. Maintaining work and enough money for one’s own needs were found to be significant protective factors of depression (OR 0.37 and 0.29, respectively). Conclusions: Exacerbation of insomnia and depression during the pandemic needs to be addressed. Economic crisis seems to influence mental state even more than COVID diagnosis among study subjects/relatives.

## 1. Background

According to data from the World Health Organization (WHO), over 145,000,000 infections of SARS-CoV-2 have been confirmed worldwide, with the majority of cases from the United States [1]. In Poland, the most recent data report over 2,700,000 cases and more than 64,000 deaths [2].

Global attention has been drawn to the health consequences of patients who become infected with SARS-CoV-2. However, important social problems, such as mental disorders, also need to be diligently addressed in the face of this crisis. This topic is particularly important given that the COVID-19 pandemic has led to limitations in essential medical care, including care in the fields of psychiatry and psychotherapy [3]. Psychiatric patients, as well as members of the general population, are at increased risk for many mental health problems, leading to impaired function and poorer cognition [4]. Some examples of reasons for mental health deterioration can include fear of the disease, fear for the safety of loved ones, uncertainty of the future, and also economic reasons—such as job and money loss. Reports from China clearly show that the COVID-19 epidemic poses a parallel threat in the form of an epidemic of depression [3]. The present study was designed to confirm this phenomenon. In the face of stress that affects people around the globe, healthcare systems must be prepared for such a mental epidemic due to the COVID-19 pandemic. In particular, social distancing is considered to be the most efficient way to reduce the risk of COVID-19. At the same time, access to medical care professionals has become substantially reduced, which reduces patients’ access to appropriate treatment [3]. On the other hand, the worsening mental states of untreated patients can lead to individuals ignoring the regulations of social distancing and related precautions. Poor adherence to social distancing can further exacerbate the viral epidemic, as has already been reported in several countries (e.g., China). For example, in a study of psychiatric hospitals in Wuhan, China, SARS-CoV-2 infections have been noted among 50 patients and 30 healthcare workers [5].

A statistical report from Poland found that 3–7% of individuals in the general population currently suffer from a depressive disorder. The authors of the report underscored that this number may even be an underestimation, which may be due to poorer survey responses from respondents with mental health problems [6]. Nevertheless, we can assume that, including other mental conditions (e.g., bipolar, anxiety or substance abuse/dependence), up to 25% of individuals in the Polish population will require psychiatric care at some point in their life [6]. Chronic stress and insecurity induced on the global stage by the SARS-CoV-2 pandemic can exacerbate the misuse of anxiolytics, especially benzodiazepines [7].

Stress, insomnia, and depression are strongly correlated in the field of psychopathology. Acute and chronic stress influence the metabolic, cardiovascular, and immune systems [8]. Neural circuitry under physiological conditions easily adapts to a new situation [9]; however, when stress becomes chronic, maladaptive processes can occur. When stressful circumstances persist, excessive activation of brain excitatory pathways can lead to a disruption in brain function, further potentiated by glucocorticoids [10,11,12,13,14,15,16]. Stress plays an important role in neuronal remodeling and leads to further negative psychiatric and psychological consequences [17,18,19,20,21,22,23,24]. As much research has proven, chronic stress has been linked to both depression and insomnia [25,26,27] and several mechanisms that connect these effects have been reported [28,29,30,31,32,33,34,35].

Limiting social contact and social distancing will probably be recommended further in subsequent months or even years. It can be expected that the negative psychological consequences of isolation will emerge in the coming years. Social support plays a pivotal role in mood regulation among humans. In current times, social support is particularly needed though very difficult to obtain [36]. A pandemic crisis brings with it a lot of fear, pressure and stress, and in such circumstances, people tend to unite with and support each other. The fact that the current crisis is caused by an infectious disease makes it very difficult to receive support from a face-to-face relationship.

## 2. Objectives

The present study aimed to test whether symptoms of depression and insomnia were exacerbated by the COVID-19 pandemic. The second goal was to investigate changes in social support received during the pandemic. To accomplish these, we assessed current symptoms of depression and insomnia during COVID-19 and compared them to levels reported prior to the outbreak of the pandemic. We also assessed social support during the pandemic and compared it to the level reported prior to the beginning of the pandemic. Our hypothesis was that significant changes in the mental states of patients would be found. While the Polish authorities were continuing to announce further restrictions, we explored the impact of these conditions on subjects’ mental states. Further, we sought factors related to changes in mental state. We tested if changes in symptoms of depression and insomnia were associated with the following: age, gender, education status, living alone vs. being in a relationship, job conditions, income, and patients’ and their relatives’ COVID-19 diagnoses.

## 3. Methods

The study was conducted from 1 April to 15 May 2020, on a group of psychiatric patients and healthy controls.

Patients were registered in a psychiatric outpatient clinic in Warsaw, Poland, and had a diagnosis of somatoform, neurotic or stress-related disorders as well as depression, according to the ICD-10 [37]. Potential eligible patients also had a psychiatric consultation during the last year. Potential participants were invited to participate in the study via an email invitation. Patients’ email addresses were obtained from the clinic’s database, using the most recent email address provided by patients during the registration process. The email invitation provided crucial information about the subject of the study, its purpose, and expected results. The patient’s role in the study was clearly described. Among the 240 email invitations sent out, a total of 212 patients decided to take part in the study and returned properly completed questionnaires. The refusal rate was 11.7%. If a patient consented to participate in the study, the ability to complete the online survey was enabled. Thus, the patient group (P) consisted of 212 individuals. A control group (C), consisting of 207 participants, volunteered to be a part of the study and were matched with the study group. The first 20 volunteers were directly asked by authors to participate in the study. This group provided referrals using social connections, and the control group was recruited using this snowball effect. Participants in this group were asked if they had been treated for any psychiatric disorder and if so, what was the diagnosis. Individuals who reported receiving psychiatric care were moved to the patient group. The data were analyzed in a manner that ensured the subjects’ anonymity. The study protocol was prepared according to the Declaration of Helsinki, and was approved by IRB at the Medical University of Warsaw.

Informed consent was provided by participants by marking a box, in which participants declared that they understood all the information and agreed to participate in the study. The questionnaire was unable to be filled and returned without giving consent.

First, subjects completed the questionnaires about the period preceding the pandemic—the last two weeks of February 2020—to examine their mental conditions prior to the announcement of the spread of the COVID-19 pandemic. Second, participants were asked the same questions regarding their current status during the last two weeks. Questions were asked in pairs (then-and-now) to enable one-to-one comparison of symptoms changing over time. Using this method, each question was answered twice (i.e., “now—in the pandemic” and “then—before pandemic started”). Questions were derived from the Beck Depression Inventory (BDI-II) [38], the Athens Insomnia Scale (AIS) [39], and the Medical Outcomes Study Social Support (MOS-SSS) [40]. The pre-pandemic assessment time—the last two weeks of February 2020—was established arbitrarily. Mental state prior to the pandemic was not possible to test in real time, given that the rapid spread of COVID-19 was unanticipated. Pre- and post-pandemic assessments were conducted at the same time. Longitudinal evaluation of mental health symptoms was not performed because it was not possible due to the unexpected nature of the COVID-19 epidemic. Thus, the subjects were asked to assess their mental state during the pre-pandemic period with maximal accuracy. None of the study’s subjects reported difficulties with recalling that period. Participants were also asked whether they or their relatives were diagnosed with a SARS-CoV-2 infection.

## 4. Measures

The questionnaire comprised the following scales:

1. The Beck Depression Inventory (BDI-II) was used to evaluate depressive symptoms. The BDI-II consists of 21 questions. There are four variants of response corresponding with the increasing intensity of symptoms, scoring from 0 to 3. The Polish adaptation of the scale by Łojek and Stańczak was used with Cronbach’s alpha = 0.91. The higher the score, the greater the depressive symptoms. Cut-off scores for the BDI-II are: 0–13 points—minimal range; 14–19 points—mild depression; 20–28 points—moderate depression; 29–63 points—severe depression [38];
2. The Athens Insomnia Scale (AIS) was used to evaluate sleep disorders. Higher scores correspond with more severe symptoms of insomnia. Scores of 0–5 points are considered to be in the normal range. Scores of 6 points or higher indicate sleep disorders. The Polish adaptation of the scale with Cronbach’s alpha = 0.9 was used [39].
3. The Social Support Scale (MOS-SSS) was used to evaluate social support. Higher scores indicate respondents having higher rates of social support. The scoring was as follows: 19–38 points—low score; 38–76 points—medium score; 76–95 points—high score. Cronbach’s alpha for the scale was as high as 0.97 [40].

Cronbach’s alpha for all scales used in this study was higher than 0.7. Therefore, this study can be considered as reliable.

Demographic data along with employment and financial status were also collected. Patients were also asked if themselves or their relatives had been diagnosed with COVID-19, which may be the pivotal factor in altering subjects’ mental conditions.

Statistical analyses were conducted using the SPSS Statistics 25 Package by IBM. The selection of the appropriate test was made on the basis of the homogeneity of variance in the compared groups. Due to the non-normal distribution of variables and the different number of participants among the examined groups, non-parametric tests were used when correlations were considered. First, a comparison of depression and insomnia severity was performed between two analyzed moments in time. Furthermore, we analyzed the correlation of factors, such as age, gender, education, living alone vs. being in a relationship, employment status, income, individuals’ and their relatives’ COVID-19 diagnoses, and social support, with an increase in insomnia and depression. To check whether there are statistically significant differences between the two time periods for the analyzed variables, the Wilcoxon test was used. The analysis of the Spearman correlation allowed us to check the presence of a statistically significant relationship between the studied variables. Then, a multinomial logistic regression was performed against the factors that were significantly correlated with the increase in symptoms of depression and insomnia.

These analytical methods were chosen because the groups to be compared were unequal or small. Additionally, for this reason, non-parametric tests were used. This was also due to the abnormal distribution of the examined variables. Logistic regression analysis showed no statistically significant predictors other than those described in the outcomes. No other examined factors influenced the remaining results of the tested scales.

The severity of insomnia and depression as well as social support were gathered and analyzed as continuous variables. Working status and change in income were binary variables.

## 5. Results

The patients group consisted of 147 women and 65 men. The mean age was 36 years (19–74). A total of 100 participants were single (single, divorced, widowed), and 112 were in a relationship (unmarried or married). The control group consisted of 143 women and 64 men. The mean age was 35 years (19–74). A total of 84 participants were single (single, divorced, widowed), and 123 were in a relationship (unmarried or married).

The COVID-19 pandemic resulted in the aggravation of depressive symptoms and insomnia in both patient and control groups. The level of social support decreased during the pandemic in patients but increased among controls. These differences presented a statistical significance. In particular, in the group of patients, the mean MOS-SSS during the pandemic was significantly lower (indicating lower social support) as compared to the MOS-SSS scores prior to the pandemic period (Z = 5.83; *p* < 0.001). The opposite pattern was observed in the control group (Z = 8.51; *p* < 0.001).

For the Athens Insomnia Scale (AIS), the mean of this variable in both groups was statistically significantly lower (indicating lower insomnia) before the pandemic compared to during the pandemic period (patients: Z = 2.96; *p* = 0.003, controls: Z = 3.32; *p* = 0.001). A similar pattern was observed for the Beck Depression Inventory–II (BDI II), wherein mean depressive scores were significantly lower before the pandemic as compared to the pandemic period (patients: Z = 6.08; *p* < 0.001, controls: Z = 8.69; *p* < 0.001).

Changes in social support, (MOS-SSS), insomnia (AIS), and depressive symptoms (BDI) between the pre-pandemic and pandemic periods are presented in Table 1.

Next, the same analysis was performed to test for differences over time among the gender groups (i.e., in males and females). Similar differences were obtained in terms of MOS-SSS and scores changed over time in both males and females. Importantly, for the AIS scores, only women showed differences over time, across both P and C groups (Z = 2.47; *p* = 0.01 and Z = 2.79; *p* = 0.005, respectively).

In particular, the AIS scores reported prior to the pandemic were significantly lower as compared to those reported during the pandemic period. The most substantial changes were observed for the BDI scores, such that depressive scores were higher during the pandemic as compared to before the pandemic. This difference reached significance in both the male and female groups. Results are presented in Table 2.

We also investigated factors that may correlate with the observed increase in symptoms of depression or insomnia. All the statistically significant correlations are discussed and described below.

### 5.1. Patients’ Significant Outcomes

Among patients, work status (i.e., whether the person is working or not) was significantly associated with the change in depressive scores from the pre-pandemic period to during the pandemic period. A significantly higher median difference in depressive scores between the two time periods was observed among patients who did not work as compared to those who did work during the pandemic (Table 3).

For the BDI scores, a logistic regression analysis was used to test what factors predict an increase in BDI scores. In this model, work status (working or not) and money for one’s needs (enough or not enough money for living) were significant predictors of an increase in BDI scores in the patients group. An increase in BDI scores was predicted by non-work status and not having enough money for one’s own needs: performed work with OR = 0.37; 95% CI = 0.19–0.97; *p* = 0.008; enough money for needs with OR = 0.29; 95% CI = 0.1–0.82; *p* = 0.02.

### 5.2. Control Group’s Significant Outcomes

We also studied the influence of a decrease in income in the pandemic on mental state. An increase in income was not considered given that only a small number of individuals reported an increase. In the control group (but not among patients), those who have experienced a decrease in income in the pandemic reported a greater increase in BDI depression scores (Table 4).

In the control group, age was negatively correlated with BDI scores during the pandemic, r = −0.19; *p* = 0.003. In particular, the younger the person, the higher the BDI score during the pandemic.

In the control group, there was also a significant correlation between education and age with the change in AIS scores over time. Higher education and older age were associated with a greater increase in symptoms of insomnia during the pandemic as compared to prior the pandemic; r = 0.18; *p* = 0.01 and r = 0.15; *p* = 0.03, respectively.

No correlation between other analyzed factors with insomnia was observed.

Social support before and during the pandemic (r = -0.23; *p* = 0.003) was significantly lower among older individuals as compared to younger individuals in the control group.

No other factors were significantly correlated with symptoms of insomnia or depression, including age, gender, education, living status (i.e., alone vs. in relationship), work situation, one’s and/or a relative’s diagnosis of COVID-19 (or death).

Surprisingly, there was also no association between depression and social support in either group.

## 6. Discussion

Depressive symptoms, measured with the BDI-II questionnaire, were exacerbated among both the patient and control groups. This increase in depressive symptoms may be explained by stress-related mechanisms. Similarly, the outcome of the Athens Insomnia Scale showed an increase in insomnia in both the patient and control groups. Furthermore, there were interesting correlations between depressive symptoms, insomnia, and factors, such as: age, gender, education, employment, and resources for one’s needs. For insomnia, female gender was associated with greater symptoms as compared to male gender. This gender difference is in line with the occurrence of insomnia in the general population, where sleep disorders are about 1.5 times more common among women than men [41]. The greater increase in depressive symptoms during the pandemic was primarily driven by controls who experienced a decrease in income during the epidemic. These findings should be factored into psychiatric treatment, given that low socioeconomic status and poverty are important risk factors for suicide [42]. Factors correlated with increasing depressive symptoms were further investigated. We found that employment status (i.e., whether the person is working or not) was a significant predictor of change in depressive symptoms. Unemployment is another well-known risk factor for suicide, especially at the very beginning of unemployment [43]. In our sample, higher education was linked to an increase in symptoms of insomnia during the pandemic. This finding is in opposition to a study by Garland et al., who reported no significant link between education level and insomnia [44]. It is possible that controls with higher education levels had more knowledge about SARS-CoV-2, and more fear due to the epidemic. In the control group, older age was associated with a lower increase in BDI scores during the pandemic, but prior to the pandemic, there was no association between age and BDI scores. In Poland, younger people may be more serious about the restrictions and may be more likely to wear masks and/or maintain social distance as compared to older individuals. Based on our observations here in Warsaw, everyday attitudes of older Polish people may be more careless. Therefore, one of the possible explanations of lower depressive symptoms among older individuals is that older people may not be as terrified of the virus as younger individuals.

Our study revealed that an individual’s income and employment status can be important factors linked with depressive symptoms. These findings are especially important when a global economic crisis becomes a reality. After the onset of COVID-19, millions of employees lost their places of work. This may suggest that even more people will fall into poverty [45]. The OECD published data stating that between the last three months of 2019 and the second quarter of 2020, the OECD-wide GDP was projected to have fallen by almost 15%. In addition, the hours worked fell as much as ten times more compared to the first quarter of the financial crisis in 2008 [43]. Accordingly, maintaining employment and financial support for individuals may be crucial for reducing the mental health impacts of the COVID-19 pandemic. A future-planned phase of this study will examine participants about six months after the pandemic has ended.

## 7. Limitations

The main limitation of this study is that the data collected were self-reported. The authors understand that asking participants to recount their mental state in the past can yield some bias. However, in our study, the patients’ perceptions of alterations in their mental conditions are crucial. As noted previously, all subjects completed the survey by responding to the same question twice (in a then-and-now fashion), which corresponded to their present (i.e., during the pandemic) mental state and their mental state before the pandemic. However, this method of gathering information is in the nature of psychiatric examination. Psychiatrists evaluate symptoms, mostly using a patient’s self-report. At this time, there is no fully objective measure that can be used to evaluate depressive symptoms free from a patient’s subjective perspective. Thus, a patient’s impression of the worsening or improvement of their mental condition remains the cornerstone for therapeutic evaluations. However, the main limitation of the research is that pre-pandemic and post-pandemic outbreak assessments have been conducted simultaneously in this study.

Additionally, the cross-sectional nature of this study, which was based on two assessments carried out at the same moment in time, can result in bias. Such an approach is clearly less relevant than the assessment that would be performed at two different points of time. A study with a longitudinal design would be more precise and feasible, but unfortunately, the course of the pandemic did not allow for that. Thus, many origins of bias might have influenced the pre-epidemic perceptions of the subjects’ mental states. This can be, for instance, problems remembering, the impact of current stress, and fear for the future. All aforementioned factors should be taken into consideration while interpreting the outcomes. Typically for cross-sectional studies, when the outcome and exposure are measured at the same time, establishing causal relationships is relatively difficult.

Thus, authors need to stress that given the cross-sectional and retrospective design of this study, the outcomes should be interpreted with caution.

## 8. Conclusions

This study, assessing the pandemic’s influence on human mental state, is of great significance. It provides important information, in both the fields of research and clinical medicine. Investigating the reasons and correlations of psychiatric symptoms could help to elucidate the mechanisms underlining insomnia and depressive disorders on clinical grounds. This, in turn, may lead to new ideas about how to modify and improve methods to diagnose and treat depression and insomnia. Deterioration of mental health with the exacerbation of depression and insomnia during the COVID-19 pandemic is a scientific fact and needs to be addressed by health care policies.

## Figures and Tables

**Table 1:** MOS-SSS, AIS, and BDI scores reported during the pre-pandemic period and during the pandemic across groups. Wilcoxon test.

	PATIENTS Pre-Pandemic Period Mean Median SD	PATIENTS Current Period Mean Median SD	*p*	Z	CONTROLS Pre-Pandemic Period Mean Median SD	CONTROLS Current Period Mean Median SD	*p*	Z
MOS-SSS	71.85 74.5 18.59	68.31 70 19.4	**>0.001**	5.83	74.7 76 17.87	79.76 83 17.2	**>0.001**	8.51
AIS	3.88 3 3.22	4.54 4 3.31	**=0.003**	2.96	2.91 2 2.3	3.46 3 2.67	**=0.001**	3.32
BDI	14.65 11 13.9	20.33 19 14.9	**>0.001**	6.08	5.7 3 7.5	11.3 9 9.72	**>0.001**	8.69

**Table 2:** MOS-SSS, AIS, and BDI scores during the pre-pandemic period and during the pandemic across groups, by gender. Wilcoxon test.

	Female Patients Pre-pandemic Period Mean Median SD	Female Patients Current Period Mean Median SD	Z *p*	Male Patients Pre-pandemic Period Mean Median SD	Male Patients Current Period Mean Median SD	Z *p*	Female Controls Pre-pandemic Period Mean Median SD	Female Controls Current Period Mean Median SD	Z *p*	Male Controls Pre-pandemic Period Mean Median SD	Male Controls Current Period Mean Median SD	Z *p*
MOS-SSS	70.77 73 17.63	67.09 68 18.28	Z = 4.95; ***p* < 0.001**	74.38 81 20.58	71.15 80 21.7	Z = 3 ***p* = 0.003**	75.12 79 17.95	80.38 83 16.67	Z = 6.98 ***p* < 0.001**	73.78 76 17.79	78.39 82 18.41	Z = 4.89 ***p* < 0.001**
AIS	4.1 3 3.25	4.77 4 3.2	Z = 2.47 ***p* = 0.01**	3.37 3 3.12	4 4 3.5	Z = 1.6 *p* = 0.11	2.92 2 2.3	3.48 3 2.71	Z = 2.79 *p* = **0.005**	2.89 3 1.98	3.44 3 2.62	Z = 1.79 *p* = 0.07
BDI	16.19 13 14.57	21.6 19 15.26	Z = 4.47 ***p* < 0.001**	11.05 7 11.53	17.38 17 13.67	Z = 4.67 ***p* < 0.001**	5.7 2 8.2	12.24 9 10.33	Z = 7.59 ***p* < 0.001**	5.69 4 5.71	9.2 8 7.85	Z = 3.98 ***p* < 0.001**

**Table 3:** Increase of BDI from pre-pandemic to current (during pandemic) period of time among patients depending on their job status. Mann-Whitney U test.

	PATIENTS		*p*	U
	Lack of Work Performed Mean Median SD	Work Performed Mean Median SD		
Depression symptoms increase (measured as BDI score increase)	8.01 9 13.67	4.33 3 13.36	***p* = 0.04**	U = 3896

**Table 4:** Increase of BDI from pre-pandemic to current (during pandemic) period of time among controls depending on income change during pandemic. Mann-Whitney U test.

	CONTROLS		*p*	U
	No Decrease in Income Mean Median SD	Decrease in Income Mean Median SD		
Depression symptoms increase (measured as BDI score increase)	4.05 3 7.86	8.61 7 10.16	***p* < 0.001**	U = 3251

## Data Availability

Dataset is with authors. Available on request.

## References

[1] https://www.worldometers.info/coronavirus/.

[2] https://www.gov.pl/web/koronawirus/wykaz-zarazen-koronawirusem-sars-cov-2.

[3] Liu S., Yang L., Zhang C. (2020). Mental health care in China during the COVID-19 outbreak. Lancet Psychiatry.

[4] Yang Y., Li W., Zhang Q., Zhang L., Cheung T., Xiang Y.T. (2020). Mental health services for older adults in China during the COVID-19 outbreak. Lancet Psychiatry.

[5] (2020). Collective Infections of Coronavirus among 50 Patients and 30 Health Workers in One Psychiatric Hospital in Wuhan. Shanghai Obs. https://www.jfdaily.com/news/detail?id=208584.

[6] Kiejna A., Adamowski T., Piotrowski P., Moskalewicz J., Wojtyniak B., Świątkiewicz G., Stokwiszewski J., Kantorska-Janiec M., Zagdańska M., Kessler R.C. (2015). Epidemiologia zaburzeń psychiatrycznych i dostępność psychiatrycznej opieki zdrowotnej. EZOP—Polska. Psychiatr. Pol..

[7] Panes A., Fourrier-Réglat A., Verdoux H., Tournier M. (2018). Use and misuse of benzodiazepines in patients with psychiatric disorders. Presse Med..

[8] McEwen B.S. (2017). Neurobiological and Systemic Effects of Chronic Stress.

[9] Gray J.D., Rubin T.G., Hunter R.G., McEwen B.S. (2014). Hippocampal gene expression changes underlying stress sensitization and recovery. Mol. Psychiatry.

[10] McEwen B.S., Weiss J., Schwartz L. (1968). Selective retention of corticosterone by limbic structures in rat brain. Nature.

[11] Reul J.M., DeKloet E.R. (1985). Two receptor systems for corticosterone in rat brain: Micro distribution and differential occupation. Endocrinology.

[12] McEwen B.S. (2016). Stress-induced remodeling of hippocampal CA3 pyramidal neurons. Brain Res..

[13] Bennur S., Rao B.S., Pawlak R., Strickland S., McEwen B.S., Chattarji S. (2007). Stress-induced spine loss in the medial amygdala is mediated by tissue-plasminogen activator. Neuroscience.

[14] Lau T., Bigio B., Zelli D., McEwen B.S., Nasca C. (2017). Stress-induced structural plasticity of medial amygdala stellate neurons and rapid prevention by a candidate antidepressant. Mol. Psychiatry.

[15] Vyas A., Mitra R., Rao B.S., Chattarji S. (2002). Chronic stress induces contrasting patterns of dendritic remodeling in hippocampal and amygdaloid neurons. J. Neurosci..

[16] Rao R.P., Anilkumar S., McEwen B.S., Chattarji S. (2012). Glucocorticoids protect against the delayed behavioral and cellular effects of acute stress on the amygdala. Biol. Psychiatry.

[17] Radley J.J., Sisti H.M., Hao J., Rocher A., McCall T., Hof P.R., McEwen B.S., Morrison J.H. (2004). Chronic behavioral stress induces apical dendritic reorganization in pyramidal neurons of the medial prefrontal cortex. Neuroscience.

[18] Popoli M., Yan Z., McEwen B.S., Sanacora G. (2012). The stressed synapse: The impact of stress and glucocorticoids on glutamate transmission. Nat. Rev. Neurosci..

[19] Revollo J.R., Cidlowski J.A. (2009). Mechanisms generating diversity in glucocorticoid receptor signaling. Ann. N. Y. Acad Sci..

[20] Hill M.N., McEwen B.S. (2010). Involvement of the endocannabinoid system in the neurobehavioural effects of stress and glucocorticoids. Prog. Neuro-Psychopharm. Biol. Psychiat..

[21] Wei Q., Lu X.Y., Liu L., Schafer G., Shieh K.R., Burke S., Robinson T.E., Watson S.J., Seasholtz A.F., Akil H. (2004). Glucocorticoid receptor over-expression in forebrain: A mouse model of increased emotional lability. Proc. Natl. Acad. Sci. USA.

[22] Zohar J., Yahalom H., Kozlovsky N., Cwikel-Hamzany S., Matar M.A., Kaplan Z., Yehuda R., Cohen H. (2011). High dose hydrocortisone immediately after trauma may alter the trajectory of PTSD: Interplay between clinical and animal studies. Eur. Neuropsychopharmacol..

[23] Schelling G., Roozendaal B., De Quervain D.J.F. (2004). Can posttraumatic stress disorder be prevented with glucocorticoids?. Ann. N. Y. Acad Sci..

[24] Mitra R., Sapolsky R.M. (2008). Acute corticosterone treatment is sufficient to induce anxiety and amygdaloid dendritic hypertrophy. Proc. Natl. Acad. Sci. USA.

[25] Gold P.W., Goodwin F.K., Chrousos G.P. (1988). Clinical and biochemical manifestations of depression: Relation to the neurobiology of stress II. N. Engl. J. Med..

[26] Goodyer I.M., Herbert J., Tamplin A., Altham P.M.E. (2000). Recent life events, cortisol, dehydroepiandrosterone and the onset of major depression in high-risk adolescents. Br. J. Psychiatry.

[27] Finlay-Jones R., Brown G.W. (1981). Types of stressful life event and the onset of anxiety and depressive disorders. Psychol. Med..

[28] Hammen C. (2005). Stress and depression. Annu Rev Clin Psychol..

[29] Abramson L.Y., Alloy L., Hankin B., Haeffel G., MacCoon D., Gibb B. (2002). Cognitive vulnerability-stress models of depression in a self-regulatory and psychobiological context. Handbook of Depression.

[30] Bifulco A., Brown G.W., Moran P., Ball C., Campbell C. (1998). Predicting depression in women: The role of past and present vulnerability. Psychol. Med..

[31] Breslau N., Davis G.C. (1986). Chronic stress and major depression. Arch. Gen. Psychiatry.

[32] Davila J., Bradbury T.N., Cohan C.L., Tochluk S. (1997). Marital functioning and depressive symptoms: Evidence for a stress generation model. J. Personal. Soc. Psychol..

[33] Bifulco A., Bernazzani O., Moran P.M., Ball C. (2000). Lifetime stressors and recurrent depression: Preliminary findings of the Adult Life Phase Interview (ALPHI). Soc. Psychiatry Psychiatr. Epidemiol..

[34] Ensel W.M., Lin N. (1996). Distal stressors and the life stress process. J. Community Psychol..

[35] Garnefski N., Van Egmond M., Straatman M. (1990). The influence of early and recent life stress on severity of depression. Acta Psychiatr. Scand..

[36] Brown G.W., Harris T.O. (1978). Social Origins of Depression.

[37] World Health Organisation (2009). International Statistical Classification of Diseases and Related Health Problems.

[38] Beck At Ward Ch Mendelson M., Mock J., Erbaugh J. (1961). An inventory for measuring depression. Arch Gen Psychiatry.

[39] Soldatos C.R., Dikeos D.G., Paparrigopoulos T.J. (2000). Athens Insomnia Scale: Validation of an instrument based on ICD-10 criteria. J. Psychosom. Res..

[40] Sherbourne C.D., Stewart A.L. (1991). The MOS social support survey. Soc. Sci. Med..

[41] Suh S., Cho N., Zhang J. (2018). Sex Differences in Insomnia: From Epidemiology and Etiology to Intervention. Curr. Psychiatry Rep..

[42] Iemmi V., Bantjes J., Coast E., Channer K., Leone T., McDaid D., Palfreyman A., Stephens B., Lund C. (2016). Suicide and poverty in low-income and middle-income countries: A systematic review. Lancet Psychiatry.

[43] Milner A., Page A., LaMontagne A.D. (2013). Long-term unemployment, and suicide: A systematic review and meta-analysis. PLoS ONE.

[44] Garland S.N., Barg F.K., Cakouros B., Gehrman P., DuHamel K.N., Mao J.J. (2018). A qualitative examination of the factors related to the development and maintenance of insomnia in cancer survivors. Palliat. Supportive Care..

[45] OECD (2020). OECD Employment Outlook 2020: Worker Security and the COVID-19 Crisis.

